# In vitro biotransformation of pyrrolizidine alkaloids in different species. Part I: Microsomal degradation

**DOI:** 10.1007/s00204-017-2114-7

**Published:** 2017-11-16

**Authors:** Franziska Kolrep, Jorge Numata, Carsten Kneuer, Angelika Preiss-Weigert, Monika Lahrssen-Wiederholt, Dieter Schrenk, Anja These

**Affiliations:** 1 German Federal Institute for Risk Assessment, Max-Dohrn-Straße 8-10, 10589 Berlin, Germany; 2 University of Kaiserslautern, Food Chemistry and Toxicology, Erwin-Schrödinger-Straße 52, 67663 Kaiserslautern, Germany

**Keywords:** Pyrrolizidine alkaloids, In vitro metabolism, Species, Mass spectrometry

## Abstract

Pyrrolizidine alkaloids (PA) are secondary metabolites of certain flowering plants. The ingestion of PAs may result in acute and chronic effects in man and livestock with hepatotoxicity, mutagenicity, and carcinogenicity being identified as predominant effects. Several hundred PAs sharing the diol pyrrolizidine as a core structure are formed by plants. Although many congeners may cause adverse effects, differences in the toxic potency have been detected in animal tests. It is generally accepted that PAs themselves are biologically and toxicologically inactive and require metabolic activation. Consequently, a strong relationship between activating metabolism and toxicity can be expected. Concerning PA susceptibility, marked differences between species were reported with a comparatively high susceptibility in horses, while goat and sheep seem to be almost resistant. Therefore, we investigated the in vitro degradation rate of four frequently occurring PAs by liver enzymes present in S9 fractions from human, pig, cow, horse, rat, rabbit, goat, and sheep liver. Unexpectedly, almost no metabolic degradation of any PA was observed for susceptible species such as human, pig, horse, or cow. If the formation of toxic metabolites represents a crucial bioactivation step, the found inverse conversion rates of PAs compared to the known susceptibility require further investigation.

**Electronic supplementary material:**

The online version of this article (10.1007/s00204-017-2114-7) contains supplementary material, which is available to authorized users.

## Introduction

Pyrrolizidine alkaloids (PAs) are secondary metabolites of certain flowering plants and have long been known to cause acute and chronic toxicity in humans. PAs became known more than hundred years ago when they mainly presented a problem for animal health, but recent findings of relatively high PA amounts in tea and herbal infusions revealed that contaminations with PAs are also a relevant topic for food safety (BfR 2013; Bodi et al. 2014). Historically, PA containing plants or their ingredients were recognized as poisonous, because after their ingestion, they cause acute toxic effects and the occurring symptoms can be directly linked to the ingested plant. Poisoning incidents with species of *Crotalaria* as well as *Senecio* were described to cause extensive loss of livestock in many countries (Armstrong and Zuckerman 1970; Hill 1960; Selzer and Parker 1951; Stuart and Bras 1956; Theiler 1919, 1920). As both genera belong to botanically unrelated plant families (*Crotalaria* to Fabaceae and *Senecio* to Asteraceae), it was not clear from the beginning that the same group of secondary metabolites was responsible for the adverse effects. Later, a particular form of infantile liver cirrhosis was reported to occur endemically in Jamaica (Bras and Hill 1956; McFarlane and Branday 1945). The clinical sign was described as veno-occlusive disease (VOD) and similarities in the hepatocellular damage after *Senecio* and *Crotalaria* poisoning were recognized. Based on the performance of animal tests, the VOD cases in Jamaica were traced back to *Crotalaria* plants consumed as “bush teas”; however, *Senecio* plants appeared to play a similar role (Bras et al. 1957). Independently, comparable clinical features were recognized during large outbreaks of VOD in Afghanistan and Uzbekistan caused by a contamination of grain with *Heliotropium* plants, which in turn belong to the Boraginaceae (Datta et al. 1978; Mohabbat et al. 1976; WHO 1988).

The acute toxic potential of PAs observed after consumption of food or feed contaminated with PA containing plants was later confirmed by controlled animal experiments (Cheeke 1988; Mattocks 1986; Roeder 1995, 2000; Wiedenfeld and Edgar 2011). Acute poisoning with PAs was linked to a limited number of plant species, even though other species of the same plant families are also known to have PA-producing capacities. For instance, all genera of Boraginaceae are supposed to synthesize PAs, whereas reported cases of intoxication are mainly related to *Heliotropium* species. The findings suggest that there is not only a relation between toxicity and the total PA content but also a dependence on the PA profile. Recent attempts to assess the risk of different PA congeners from the available literature on in vivo data for acute toxicity and in vitro data for genotoxic effects led to the derivation of interim relative potency factors for a number of abundant PAs (Merz and Schrenk 2016).

**Fig. 1:**
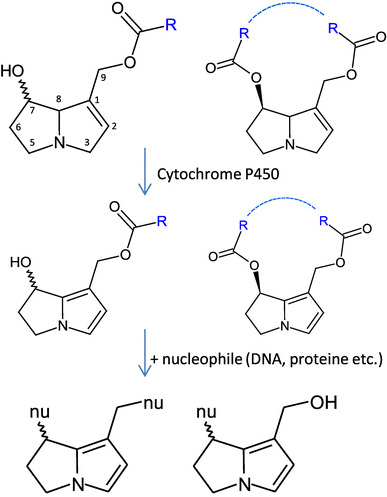
Structures of PAs and selected metabolic pathways proposed for acute toxicity and genotoxic effects (Fu et al. 2004; Mattocks 1968)

The thorough screening of plants revealed that mainly, plant families of Boraginaceae, Asteraceae, and Fabaceae produce PAs, and although they are found worldwide, those plants growing in warmer climates often showed to have higher alkaloid levels or are likely to thrive following periods of drought (Mattocks 1986). Total PA contents are known to vary between trace amounts and 20% based on dry mass (Johnson et al. 1985; These et al. 2013). Although the structures formed by respective plant families differ, all of the individual hepatotoxic PAs are esters of l-hydroxymethyl-1,2-dehydro-7-hydroxy-pyrrolizidines (Fig. 1). This necine base is esterified at one or both hydroxy groups, i.e., at the C7 and/or C9 positions with the so-called necic acids forming mono- or diesters (Fig. 1). Those diesters can be open chained or cyclic, and in addition, a subgroup of cyclic diesters—the otonecines—consists of an azacyclooctene ring system that is methylated at the *N*-atom (Fig. 1).

Animal tests performed with isolated individual PAs confirmed that the toxicity of respective plants is not caused by a single PA congener. Acute toxicity was determined for several PAs administered by intraperitoneal or intravenous injection in mice and rats. Although marked variations in the toxicity were observed, almost all PAs were able to cause characteristic, mostly hepatic effects (Bull et al. 1958; Culvenor et al. 1969; Mattocks 1972; Schoental 1968, 1970). This suggests that the pyrrolizidine core structure common to all PAs represents the toxicologically relevant moiety.

It is generally accepted that PAs themselves are biologically inactive and require metabolic activation to exert their toxic effects (Chen et al. 2016; Fashe et al. 2015; Fu et al. 2004; He et al. 2016; Mattocks 1986; Mei et al. 2010; Roeder 2000; Wiedenfeld and Edgar 2011). The non-toxic metabolites are quickly excreted, while the metabolically mediated toxification process is thought to include an oxidation to yield the corresponding dehydropyrrolizidine (pyrrolic ester) derivatives (Fig. 1) (Jago et al. 1970; Mattocks and White 1971a, b). These pyrrolic alkaloids possess an allylic structure which promotes an increase in their reactivity. Formed pyrroles are discussed as reactive alkylating agents which can rapidly bind with nucleophilic centers in DNA, proteins, amino acids, etc. and it is now believed that metabolic activation of PAs to pyrrolic ester(s), and the subsequent formation of DNA adducts is the key pathway leading to genotoxicity and carcinogenicity (Fig. 1) (Chou et al. 2003; Fu et al. 2010; Wang et al. 2005; Yang et al. 2001a; Zhao et al. 2012).

There are large species-, strain- or gender-dependent variations in the susceptibility towards PA toxicity. These observations were explained by differences in enzymatic activities or expression levels, resulting in different overall balances of the detoxification and activation pathways (Chung and Buhler 2004; Huan et al. 1998; Lin et al. 2002, 2003, 2007; Shull et al. 1976). In general, small herbivores such as sheep, goats, rabbits, hamster, and guinea pigs appear to be more resistant and tolerate higher PA dosage, while chicken and turkey, horses, cattle, and pigs are considered to be more sensitive (Anjos et al. 2010; Cheeke 1984, 1988; McLean 1970; Pierson et al. 1977; WHO 1988; Wiedenfeld and Edgar 2011). Investigations of rats revealed a moderate to high susceptibility (WHO 1988). For ruminants, the activity of rumen microbes was discussed as causing relative resistance to PA poisoning when compared to monogastric species, although the incubation of PA containing plants in sheep rumen fluid did not alter its toxicity to rats (Cheeke 1984). As the physiology of pigs including hepatic cytochromes P450 activity is generally considered to be rather similar to that of humans, the comparatively high PA susceptibility of pigs may suggest the same for humans (Hooper and Scanlan 1977; Ubiali et al. 2011).

**Fig. 2:**
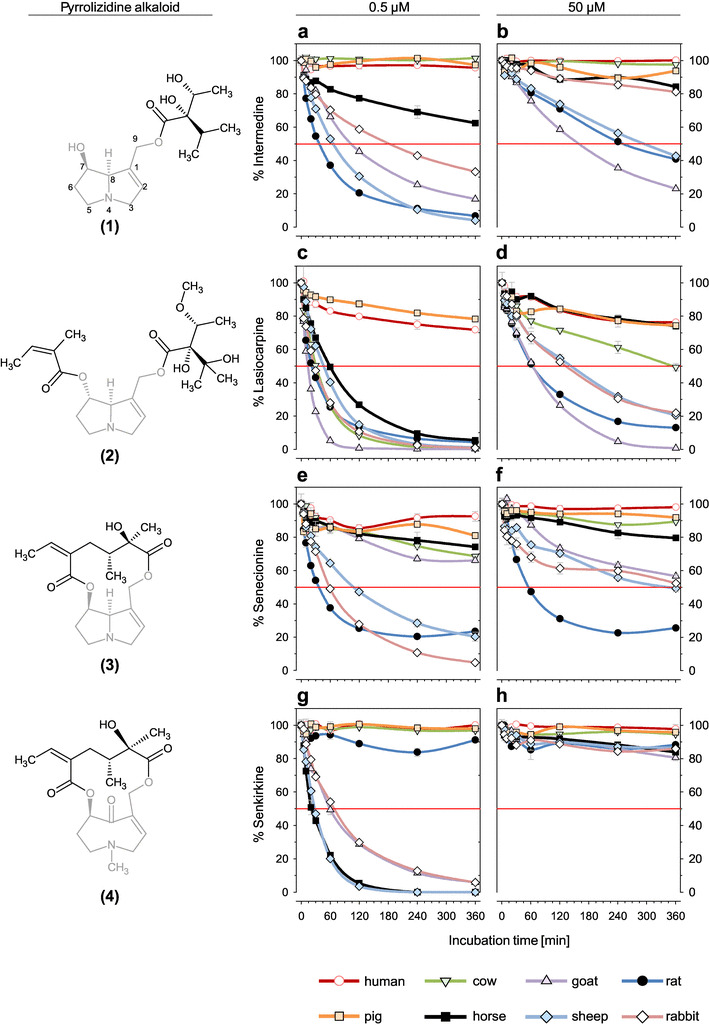
Metabolic degradation of 50 and 0.5 µM PA solutions incubated with S9 mixes from eight species as indicated, at a protein content of 1 mg/mL

The intention of our study was to compare the in vitro metabolism of a set of different PAs in liver S9 fractions from different species. We selected four of the most frequently occurring PAs representing each type of necine base and degree or type of esterification: intermedine, lasiocarpine, senecionine, and senkirkine (Fig. 2) to be examined at a higher and a lower concentration. The spectrum of species covered included human, pig, rat, rabbit, horse, cow, goat, and sheep. Due to the high number of experiments and findings, the first part of our study focused on the in vitro degradation rate.

## Materials and methods

### Chemicals and reagents

Intermedine (Im), senecionine (Sc), and senkirkine (Sk) were purchased from PhytoLab (Vestenbergsgreuth, Germany) the purity being specified as 97, 100, and 97%. Lasiocarpine (Lc) was obtained from Cfm Oskar Tropitzsch (Marktredwitz, Germany) with 98% purity. Methanol (MeOH, LC–MS grade) was purchased from Merck KGaA (Darmstadt, Germany). All other chemicals and co-factors were purchased from Carl Roth (Karlsruhe, Germany) or Sigma-Aldrich (Steinheim, Germany) at the highest purity available.

### Liver S9 fractions

Liver S9 fractions used are summarized in Table 1. All preparations were diluted in homogenization buffer to a protein content of 20 mg protein per mL S9 fraction and stored at − 80 °C until use. The homogenization buffer (pH 7.5) consisted of Tris–HCl buffer (50 mM), KCl (150 mM), and EDTA (2 mM).

**Table 1:** Characteristics of S9 fractions as provided by the supplier including own laboratory data obtained according to OECD guideline (ECVAM 2014)

Species	Strain/race	Sex	No	Supplier	Lot	Enzyme	Assay	Enzyme activity [pmol/(mg min)]
Human	Caucasian	m/f	16/16	Corning	4041007	CYP1A2	Phenacetin *O*-deethylation	240^a^
		m/f	16/16	Corning	4041007	CYP2A6	Coumarin 7-hydroxylation	240
		m/f	16/16	Corning	4041007	CYP2B6	(*S*)-Mephenytoin *N*-demethylation	7^b^
		m/f	16/16	Corning	4041007	CYP2C8	Paclitaxel 6α-hydroxylation	29
		m/f	16/16	Corning	4041007	CYP2C9	Diclofenac 4′-hydroxylation	870
		m/f	16/16	Corning	4041007	CYP2C19	(*S*)-Mephenytoin 4′-hydroxylation	7
		m/f	16/16	Corning	4041007	CYP2D6	Bufuralol 1′-hydroxylation	24
		m/f	16/16	Corning	4041007	CYP2E1	Chlorzoxazone 6-hydroxylation	360
		m/f	16/16	Corning	4041007	CYP3A	Testosterone 6β-hydroxylation	780^c^
Rat	Sprague Dawley	Male	36	Corning	4029004	CYP	Testosterone 6β-hydroxylation	970
							Testosterone 16α-hydroxylation	1000
Rabbit	New Zealand	Male	8	XenoTech	1210284		No data	
Pig	–	m/f	1/1	XenoTech	0210330		No data	
Sheep	Suffolk	Male	2	XenoTech	0410144		No data	
Goat	Angora	Female	2	XenoTech	020298B		No data	
Horse	Quarter horse	Female	1	XenoTech	0010145		No data	
Cow	No data	m/f	1/1	XenoTech	0210331		No data	

^a^16% Phenacetin degradation in 360 min for a 15 µmol

^b^37% Bupropion degradation in 360 min for a 10 µmol

^c^29% Atorvastatin degradation in 360 min for 5 µmol

### Incubation with S9 mix for phase I metabolism

An S9 mix with NADPH-regenerating system was prepared on ice with 50 mM Tris–HCl buffer, KCl (33 mM) and MgCl_2_ hexahydrate (8 mM), containing β-nicotinamide adenine dinucleotide phosphate disodium salt (NADP, final concentration 4 mM), d-glucose-6-phosphate disodium dihydrate (final concentration 5 mM), and glucose-6-phosphate dehydrogenase (final concentration 0.5 U/mL). Liver fractions were added to yield a final protein content of 1 mg/mL.

Individual PAs in a volume of 45 µL in 10% MeOH were incubated in 405 µL of S9 mix giving a final incubation volume of 450 µL and final concentration of 50 or 0.5 µM PA. Reaction mixtures were incubated at 37 °C in a Thermomixer C (Eppendorf, Germany) and reactions were terminated by adding 50 µL of the incubation mixture to a stop solution of 150 µL ice cold MeOH at different intervals (*t* = 0, 5, 10, 20, 30, 60, 120, 240, and 360 min). As a reference (*t* = 0 min), a volume of 45 µL S9 mix was added to 150 µL stop solution, and afterwards, 5 µL of PA solution were added to the stop solution to yield PA concentrations of 50 or 0.5 µM. S9 mix containing solvent only (1% MeOH) was incubated for 0 and 360 min as a blank control. To differentiate the non-metabolism related disappearance of the tested PAs, all compounds were additionally incubated without co-factors as well as without S9 fraction for 0 and 360 min. All experiments were performed in duplicate.

### Sample preparation

Samples were vortexed for 1 min and stored at − 80 °C overnight. Protein and salts were precipitated by centrifugation at 14,000×*g* for 10 min and 4 °C (centrifuge; Eppendorf, Germany). Resulting supernatants of 50 µM PA initial concentration were diluted in 5% MeOH to a final concentration of 125 nM PA. From supernatants of 0.5 µM PA initial concentration, a volume of 100 µL was withdrawn and transferred into a clean vessel containing 100 μL of water. The supernatants were evaporated to 100 µL water phase in a vacuum concentrator (Eppendorf, Germany) at room temperature to achieve a final concentration of 125 nM PA.

### Matrix calibration solution for the evaluation of PA decrease in S9 mix

For the quantification of unreacted PA substrate, an eight point matrix calibration curve was prepared for each PA covering a final concentration range from 1.25 to 150 nM. Calibration levels were prepared in the same way as the incubation samples, as described in Sect. “Sample preparation”.

### HPLC and mass spectrometry

An UltiMate 3000 (Thermo Scientific, Germany) UHPLC system was used. Reversed phase separation was achieved on a 150 × 2.1 mm; 1.9 µm C18 Hypersil Gold column with guard protection (Thermo Fisher, Runcorn, UK) at a flow rate of 0.3 mL/min. The binary mobile phase consisted of (A) 100% water and (B) 95% MeOH and 5% water both containing 0.1% formic acid and 5 mM ammonium formate. A gradient elution was adopted as follows: 0–0.5 min A:95%/B:5%, 7.0 min A:50%/B:50%, 7.5 min A:20%/B:80%, 7.6 min A:0%/B:100%, 9.0 min A: 0%/B:100%, 9.1–15 min A:95%/B:5%.

Mass spectrometry (ESI-MS/MS) data were acquired on a TSQ Quantiva (Thermo Fisher Scientific, San Jose, CA, USA). PAs were analysed in the positive ionization mode using multiple reaction monitoring (MRM). Three transitions for each PA were monitored, while the first transition was used as quantifier and the others as qualifiers Im: 300.1 → 156.1, 138.1, 120.1; Sc: 336.1 → 308.1, 138.1, 120.1 and Sk: 366.1 → 168.1, 150.1, 122.1; Lc: 412.1 → 120.1, 336.1, 220.1. A chromatogram is shown under ‘supplementary material’.

### Fitting of kinetic data

The time series of concentration data [*A*] was fitted using a linear (first order) rate law using the integrated rate equation [*A*] = [*A*]_0_ exp(− *kt*), where [*A*]_0_ is the initial concentration, *k* is the linear kinetic constant, and *t* is time. The associated half-life can be calculated as *τ* = ln(2)/*k*. Besides first order, other rate laws were also tested but discarded later (data not shown). Zero order fits yielded consistently worse goodness-of-fit *R* ^2^, where *R* is the correlation coefficient. Second order and Michaelis–Menten fits yielded somewhat better goodness-of-fit *R* ^2^ values in a few cases, but not consistently (Schnell and Mendoza 1997). Since a ranking of the apparent half-lives was the purpose of this study, a linear rate law fitting was performed on all data sets. Data were cleaned, such that concentrations determined below 10 nM were discarded, since they were less reliable. All fits were performed using in-house Matlab scripts and the Levenberg–Marquardt–Fletcher algorithm implemented as LMFnlsq (https://de.mathworks.com/matlabcentral/fileexchange/17534-lmfnlsq-solution-of-nonlinear-least-squares?requestedDomain=www.mathworks.com). Only shorter and medium half-lives which could be estimated with some degree of accuracy are given as numerical value. Half-lives estimated to be longer than 1400 min (including those for which the experiment virtually provided no change in concentration) are marked as > 1400 min.

## Results

The metabolic degradation of senkirkine, senecionine, lasiocarpine, and intermedine was detected at nine various timepoints using liver homogenates from human, pig, horse, cow, goat, sheep, rabbit, and rat. The experiments were carried out at PA starting concentrations of 50 and 0.5 µM which represent a balance between analytical sensitivity allowing a straightforward detection without any sample treatment and the exclusion of inhibitory effects of PAs towards enzymes (Fig. 2). The human S9 mix used for experiments was a pooled preparation from several donors of both sexes and the enzyme activity was certified and reported by the supplier (Table 1). In addition, we proofed the metabolic competence of the S9 mix by testing enzyme activity of CYP1A2, CYP2B6, and CYP3A4 according to OECD guidelines for testing of chemicals (Table 1) (ECVAM 2014).

**Fig. 3:**
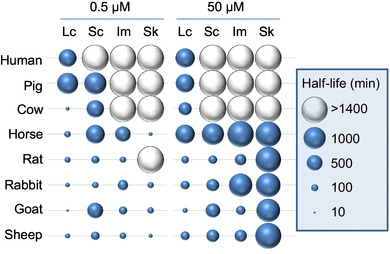
Classification of half-lives (minutes) for lasiocarpine, senecionine, intermedine, and senkirkine at a concentration of 0.5 µM (left) and 50 µM (right) for the eight investigated species

To allow a better comparison of results, the obtained data were fitted as described in 2.7 and half-lives per PA and species were estimated. The half-lives are presented in Fig. 3, and for each starting concentration, these data were arranged according to the highest and lowest values. The measured and the estimated half-live values are presented in Table 2. The numerical values and goodness-of-fit can be found in Supplementary Tables S1 and S2. For rat, goat, and sheep (and to a certain degree also for rabbit) liver S9 fractions, a substantial loss of PAs was observed for all congeners, except for senkirkine at the high starting concentration. Phase I enzymes from cow and horse liver preparations were less active in most cases.

**Table 2:** Measured (bold) and estimated (italics) PA half-life (*t*_1/2_) determination for a substrate concentration of 0.5 or 50 µM PA incubated for up to 360 min at 37 °C in S9 mix from sheep, rabbit, rat goat, horse, cow, porcine, and human

PA	[PA] in µM	*t*_1/2_ in min															
		Sheep		Rabbit		Rat		Goat		Horse		Cow		Porcine		Human	
Im	**0.5**	**66**	*80*	**183**	*210*	**37**	*80*	**103**	*130*	*	*460*	*	*#*	*	*#*	*	*#*
	**50**	**295**	*290*	*	*1070*	**252**	*260*	**160**	*170*	*	*1360*	*	*#*	*	*#*	*	*#*
Lc	**0.5**	**47**	*50*	**27**	*40*	**22**	*70*	**13**	*10*	**59**	*80*	30	*30*	*	*#*	*	*#*
	**50**	**143**	*150*	**132**	*150*	**63**	*110*	**63**	*50*	*	*720*	360	*330*	*	*#*	*	*#*
Sc	**0.5**	**108**	*140*	**60**	*80*	**37**	*120*	*	*490*	*	*690*	*	*590*	*	*#*	*	*#*
	**50**	**358**	*310*	*	*310*	**54**	*140*	*	*400*	*	*940*	*	*#*	*	*#*	*	*#*
Sk	**0.5**	**28**	*30*	**68**	*80*	*	*#*	**59**	*80*	**20**	*30*	*	*#*	*		*	*#*
	**50**	*	*1310*	*	*1170*	*	*1290*	*	*1090*	*	*1270*	*	*#*	*	*#*	*	*#*

* *t*_1/2_ > 360 min; # *t*_1/2_ > 1400 min

A striking outcome of this study was the high correlation between pig and human homogenates for all tested PAs with nearly identical metabolic degradation rates (Figs. 2, 3). Neither pig nor human preparations revealed a substantial metabolic conversion of any PA tested, although they showed a normal metabolic phase I capacity.

## Discussion

High in vitro metabolic degradation rates for a number of PAs were observed for species with low susceptibility in vivo and vice versa, i.e., the degradation rates for the most susceptible species were much lower. Under the assumption that metabolism is a bioactivation and both humans and pigs are considered as susceptible, a high conversion and, therefore, a decrease in substrate concentration could be expected. However, our findings suggest that the overall metabolic degradation of selected PAs is very low in liver S9 fractions from susceptible species. Nevertheless, highly toxic metabolites that are able to bind to proteins and enzymes may be formed in very low concentrations. This hypothesis could be supported by the fact that in vitro degradation rates for other species flatten vary slightly and do not decrease to a degradation rate of 100% (e.g., Fig. 2e).

In vitro degradation data obtained with rat S9 mix indicate a very rapid and substantial decrease in PA concentration, except for senkirkine. Comparing the metabolic degradation of PAs either incubated with rat S9 or human S9 mix very different time courses were observed (Figs. 2, 3). In the previous studies, most observations for human enzymes were obtained from microsomal preparations incubated with PAs. Studies were done for senecionine (Miranda et al. 1991), retrorsine and monocrotaline (Couet et al. 1996), riddelliine (Xia et al. 2003), and lasiocarpine (Fashe et al. 2015). The studies revealed that several metabolites are formed in liver microsomes from both human and other mammalian species, but no study monitored the overall disappearance of substrate. Therefore, the comparison of our data with results from other studies is only possible to a limited extent.

The introduction of an additional double bond into the pyrrolizidine core structure as the initial step for bioactivation is well established (Fig. 1) (Armstrong and Zuckerman 1970; Kedzierski and Buhler 1986; Schoch et al. 2000; Stegelmeier et al. 1996) and Yang et al. (2001b) demonstrated that pyrrolic metabolites are able to interact with DNA to form various adducts. Since our in vitro experiments did not reveal a substantial loss of certain PAs neither in human nor in pig S9 mix, it can be discussed if the much more pronounced metabolic loss in other species is due to detoxification or inactivation pathways, while the metabolism derived from activation plays a minor role from a quantitative point of view. In fact, even the degree of reactive pyrrolic metabolites in liver microsomes from various species was reported no to be in good correlation with the susceptibility of the species towards PAs (Huan et al. 1998). The authors suggested that rather, the species-specific balance between activation and inactivating pathways decides on the degree of toxicity. Since 1,2-saturated PAs are considered as non-toxic, the parallel investigation of PA forms which are missing the structure part essential for bioactivation might be helpful.

Referring to cow and horse, which are also regarded as sensitive species, a comparatively low metabolic conversion was revealed too (Figs. 2, 3). For the species cow only for lasiocarpine, a significant decrease was detected (half-live of 30 min, Table 2), while senecionine and senkirkine apparently were not metabolized at all (half-live of 590 and > 1400 min). Lasiocarpine is one of the main alkaloids in *Heliotropium* species that caused poisoning in cattle (Hill et al. 1997; Shimshoni et al. 2015) and DHP-derived DNA adducts were detected in beef cows accidently fed with pyrrolizidine alkaloids (Fu et al. 2017). *Senecio* spp. that form senecionine and senkirkine led to devastating intoxication in cattle, and therefore, comparable biotransformation rates as for lasiocarpine could be expected. Therefore, it seems to be a contradiction why in vitro degradation is observed for lasiocarpine and not for senecionine and senkirkine. For horse, considerable metabolic conversion was found for both lasiocarpine and senkirkine (half-live of 80 and 30 min), while the decrease of senecionine was quite low (half-live of 690 min, Table 2). Senkirkine and senecionine both occur associated in *Senecio* species which are acutely toxic to horses. Our findings indicate that overall metabolic degradation of selected PAs in liver S9 fraction does not correlate with reported toxicities in various mammalian species including humans. These data indicate that probably, detoxification pathways may vary tremendously between species and that activation pathways may play a minor role in quantitative terms although being of crucial importance for the adverse outcome. Further work is ongoing in our laboratories to identify and quantify the various metabolites formed with a focus on the balance between activating and inactivating pathways. This issue is of major importance for risk assessment of PAs in humans which are considered as being particularly sensitive towards PAs.

## Conclusions

Using liver S9 mix as a model for the study of hepatic metabolism, we demonstrated that almost no metabolic conversion was observed for any of the four investigated PA when using human or pig S9 mix. In addition, a comparatively low decrease in substrate concentration was observed for cow and horse. These species with a very low in vitro biotransformation rate were described as susceptible to PA toxicity, while for species such as rabbit, goat, or sheep that are considered as almost resistant, a much higher metabolism rate was determined (Figs. 2, 3). Considering the assumption that PAs have to undergo bioactivation for exerting toxicity, the high conversion rates for non-susceptible species seem to be in contradiction to low conversion rates in susceptible species. These observation could be explained if the observed high biotransformation rate of non-susceptible species mainly represented a detoxification and the potential of toxic metabolites that might be formed in low concentration is that high that they are able to bind to proteins and possibly inhibit S9 enzymes effectively. A quantitative assessment of all metabolites for each PA in all species should be the key to a better understanding of our findings.

Other interesting aspects of this study are the fact that horse S9 mix metabolized senkirkine but no senecionine which both occur associated in *Senecio* plants. As numerous cases of intoxication with *Senecio* plants were reported in horses, these results could be a hint that there might be differences in the toxic potential of individual PAs. Furthermore, observations for cow S9 mix, i.e., almost no decrease in concentration of *Senecio* PAs compared to a relatively high degradation of the *Heliotropium* PA lasiocarpine combined with the background that poising in cattle was observed for both plant species, might suggest that metabolic pathways for individual PAs may differ substantially. The considerable differences in in vitro biotransformation rates imply that not the overall metabolic rate but the balance between toxification and detoxification of PAs is a crucial element in the risk assessment of this group of compounds. This may have an impact not only on the general toxicity, but also on the genotoxicity and carcinogenicity of PAs in different species.

## Electronic supplementary material

Below is the link to the electronic supplementary material.

Supplementary material 1 (DOCX 53 KB)
