# Supplementary material for: In vitro biotransformation of pyrrolizidine alkaloids in different species. Part I: Microsomal degradation
Source: Arch Toxicol. 2017 Nov 16;92(3):1089–97. doi: 10.1007/s00204-017-2114-7 (PMC5866832; doi:10.1007/s00204-017-2114-7)
Supplement: Supplementary file 1 — Supplementary material 1 (DOCX 53 KB) [file 204_2017_2114_MOESM1_ESM.docx]

Supplementary material:

Extracted ion chromatograms of intermedine (orange), senecionine (blue), senkirkine (yellow) and lasiocarpine (mint green). MRM transitions used as quantifier are represented as solid lines and those used as qualifier are marked as dotted lines.
